# Effect of magnesium ion on human osteoblast activity

**DOI:** 10.1590/1414-431X20165257

**Published:** 2016-07-04

**Authors:** L.Y. He, X.M. Zhang, B. Liu, Y. Tian, W.H. Ma

**Affiliations:** 1 Department of Orthopedic Surgery, The Third Hospital of Hebei Medical University, The Key Laboratory for Orthopedic Biomechanics of Hebei Province, Shijiazhuang, Hebei Province, China; 2 Department of Orthopedic Surgery, Hebei National Defense Construction Hospital, Shijiazhuang, Hebei Province, China

**Keywords:** Magnesium, Osteoblast, Gap junction intercellular communication

## Abstract

Magnesium, a promising biodegradable metal, has been reported in several studies to increase bone formation. Although there is some information regarding the concentrations of magnesium ions that affect bone remodeling at a cellular level, little is known about the effect of magnesium ions on cell gap junctions. Therefore, this study aimed to systematically investigate the effects of different concentrations of magnesium on bone cells, and further evaluate its effect on gap junctions of osteoblasts. Cultures of normal human osteoblasts were treated with magnesium ions at concentrations of 1, 2 and 3 mM, for 24, 48 and 72 h. The effects of magnesium ions on viability and function of normal human osteoblasts and on gap junction intercellular communication (GJIC) in osteoblasts were investigated. Magnesium ions induced significant (P<0.05) increases in cell viability, alkaline phosphate activity and osteocalcin levels of human osteoblasts. These stimulatory actions were positively associated with the concentration of magnesium and the time of exposure. Furthermore, the GJIC of osteoblasts was significantly promoted by magnesium ions. In conclusion, this study demonstrated that magnesium ions induced the activity of osteoblasts by enhancing GJIC between cells, and influenced bone formation. These findings may contribute to a better understanding of the influence of magnesium on bone remodeling and to the advance of its application in clinical practice.

## Introduction

Over recent years, there has been a growing interest in magnesium and its alloys as novel internal fixation materials for bone fracture healing due to its biodegradability and mechanical properties (1-3). Magnesium implants can degrade via corrosion in the electrolytic environment of the body and mainly dissolve as divalent magnesium ions (Mg^2+^). Repeated observations have shown enhanced bone growth around various degradable magnesium alloys *in vivo*. Hong et al. (4) directly cultured a murine osteoblast-like cell line (MC3T3) on Mg-4 wt.% Zn-0.5 wt.% Zr (ZK40) alloy and found favorable cell viability and attachment. Weng et al. (5) reported that nanostructured magnesium could influence the initial adsorption of proteins known to promote the function of osteoblasts. Furthermore, a clear stimulation of cell proliferation and an enhancement of the mitochondrial respiratory activity were observed when mouse osteoblasts (MC3T3-E1) were cultured with a fluoride surface-modified AZ31 magnesium alloy (AZ31HF), compared to bare-coated ones (6). Although current studies have reported on the relationship between magnesium material and bone formation, there is still no clear understanding regarding the effects of different concentrations of magnesium ions on bone cells.

In addition, efforts have been made to reveal the mechanism by which magnesium regulates bone remodeling. Rude et al. (7) demonstrated an increase in TNF-α in bone from magnesium deficient rodents, suggesting that TNF-α may play a role in magnesium deficiency-induced bone loss. It was also reported that magnesium deficiency in the rat and/or mouse resulted in increased skeletal substance P, which in turn stimulated production of cytokines and increased bone resorption by osteoclasts (8). Moreover, Abed (9) indicated that the influx of extracellular Mg^2+^ could enhance cell migration and stimulate gene expression of melastatin-like transient receptor potential 7 (TRPM7) channels in human osteoblast MG-63 cells, leading to bone formation. Although these data support the hypothesis that magnesium ion plays a particularly critical role in bone remodeling, the exact mechanism of this biological stimulus is still unclear.

Gap junctions formed by connexins (Cx) play an important role in transmitting signals between bone cells, which are responsible for bone formation and bone remodeling. Gap junctional intercellular communication (GJIC) has been demonstrated to mediate the process of osteoblast differentiation and bone formation (10). Furthermore, GJIC propagates Ca^2+^ signaling, conveys anabolic effects of hormones and growth factors, and regulates gene transcription of osteoblast differentiation markers (10,11). To date, no information is available about the effects of magnesium ions on GJIC. Therefore, this study was performed to investigate the role of magnesium at different concentrations on osteoblast in terms of cell viability levels, alkaline phosphate (ALP) activity and osteocalcin determinations in culture supernatants. We also investigated for the first time the effects of magnesium ions on GJIC in osteoblasts.

## Material and Methods

A normal human osteoblast cell line (hFOB1.19; ATCC, USA) was used in this study. The cells were cultured in Dulbecco Modified Eagle Medium (DMEM; Gibco, USA) supplemented with 10% fetal bovine serum (Gibco), 100 IU/mL penicillin and 100 μg/mL streptomycin. Incubation was conducted at 5% CO_2_ at 37°C. The medium was changed every 2-3 days. Cells growing in medium without addition of MgSO_4_ were used as controls. MgSO_4_ was purchased from Tiancheng Pharmaceutical Co., Ltd. (China) and was diluted in serum-free DMEM to the specified concentrations.

### Cell proliferation and viability

Cell proliferation and viability were measured by the MTT 3-(4,5-dimethylthiazol-2-yl)-2,5-diphenyltetrazolium bromide assay. Cells were seeded onto a 96-well plate (clear bottom), at a density of 10,000 cells per well in 100 µL medium, incubated for 24 h, and treated with magnesium ions at final concentrations of 1, 2 and 3 mM, using 6 replicates per concentration per treatment. After 24, 48 and 72 h, 20 µL of 5 mg/mL MTT solution were added to each well and incubated in a humidified atmosphere of 5% CO_2_ at 37°C for 5 h. After incubation, the cells were washed with PBS solution. Subsequently, 100 µL of isopropanol acid 4% and hydrochloric acid were added to each well, and cells were incubated at room temperature for 10 min. The absorbance was measured by microplate reader (Synergy H4, USA) at 492 nm, with 620 nm as reference.

### ALP activity

The cells (2×10^4^ cell in 100 µL) from the second passage were seeded on four 24-well plates and incubated for 3-5 days near confluence and were treated with magnesium ions at final concentrations of 1, 2 and 3 mM. After 24, 48 and 72 h, the cells were measured for ALP activity. Analyses were performed using 6 replicates for each treatment. To assay the ALP activity, the remaining medium was removed, cells were washed with PBS and digested with 0.25% trypsin for 1 min. The cell suspension was then collected in Eppendorf tubes, distilled water was added in each tube, and the mixture was frozen in liquid nitrogen and thawed repeatedly 3-4 times to destroy the cell membrane. After centrifugation at 10,732 *g* for 15 min at -4°C, the supernatants were collected for analysis according to the ALP kit (Elabscience, China) instructions, with an automatic biochemistry analyzer (Hitachi 7150, Japan) at 405 nm. All treatments were compared against control wells (cell cultured in ordinary DMEM without magnesium).

### Osteocalcin assay

The assay for osteocalcin was performed with enzyme-linked immunosorbent assay (ELISA). The cells were cultured and treated as described above. The supernatants were collected in Eppendorf tubes to perform the osteocalcin determinations according to the instructions of the osteocalcin kit (Elabscience). The absorbance was measured by a microplate reader (Synergy H4) at 450 nm and the corresponding value of osteocalcin was calculated according to the standard curve. A total of 6 replicates were used per treatment.

### GJIC in human osteoblasts

Fluorescence recovery after photobleaching (FRAP) is a noninvasive technique that allows quantitative measurement of gap junction function in living cells. In the FRAP assay, diffusion-dependent redistribution of a gap junction-permeable fluorescent dye following photobleaching is measured. In a fluorescently labeled cell that is in contact with other labeled cells, photobleaching results in a rapid decrease in fluorescence intensity followed by fluorescence increase over a period of time due to gap junctional activity. In the present study, the FRAP was used to determine the effect of the magnesium on GJIC in normal human osteoblasts. Cells were seeded into a 96-well plate, at a density of 5,000 cells per well, and incubated to confluence. Cells were rinsed twice with D-Hank's, treated with 10 μM CFDA (5, 6-carboxy fluorescein diacetate; Sigma, USA) at 37°C for 90 min, and rinsed with D-Hank's again. Then, cells were treated with magnesium ions at final concentrations of 1, 2 and 3 mM for 24, 48 and 72 h, and their GJIC were measured with the MRC-1024 laser scanning confocal microscope imaging system (Bio-Rad, USA). The rate of fluorescence recovery (R) at 10 min after photobleaching was adopted as the functional index of GJIC.

### Statistical analysis

For each parameter of magnesium ion, mean and standard deviation for three concentrations at all time points are reported. The *t*-test was carried out to compare data between two groups and the multilevel test to compare data among three or more groups at different intervals. The SAS software package (version 9.1, SAS Institute Inc., USA) was used for statistical analysis. Statistical significance was assigned to P<0.05.

## Results

### Cell proliferation and viability

**Figure 1:**
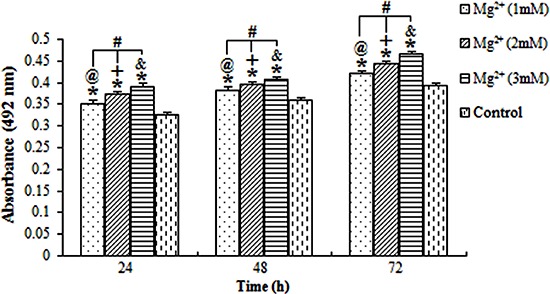
Average absorbance of osteoblasts treated with magnesium (Mg^2+^). The absorbance of cells increased with the increasing concentration of Mg^2+^ and with longer exposure time. ^@,+,&^P<0.05, differences over time at concentrations of 1, 2 and 3 mM, respectively. ^#^P<0.05, differences among various concentrations of magnesium at each time point; *P<0.05, differences from the control group at each time point (ANOVA).

The proliferation index (absorbance) of the osteoblasts exposed to magnesium ions at the concentrations of 1, 2 and 3 mM for 24, 48 and 72 were all significantly higher than those of the blank control group (P<0.05; Figure 1). Additionally, the result revealed the interaction between the exposure time and the concentration of magnesium. Data indicated that the absorbance was positively associated with the concentration of magnesium and the duration of exposure.

### ALP activity and osteocalcin level

**Figure 2:**
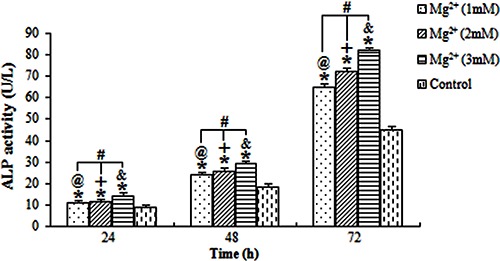
Average alkaline phosphatase (ALP) activity for osteoblastic cells treated with magnesium (Mg^2+^). The level of ALP activity for osteoblasts was stimulated by (Mg^2+^), and was positively associated with the concentration of Mg^2+^ and the time of exposure. ^@,+,&^P<0.05, differences over time at concentrations of 1, 2 and 3 mM, respectively. ^#^P<0.05, differences among various concentrations of Mg^2+^ at each time point. *P<0.05, differences from the control group at each time point (ANOVA).

**Figure 3:**
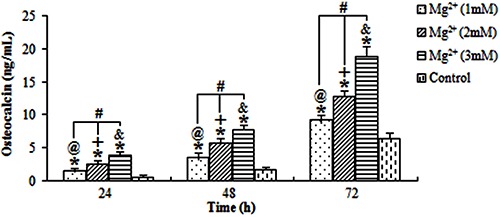
Average osteocalcin levels in osteoblasts treated with magnesium (Mg^2+^). The stimulation action of Mg^2+^ was positively associated with concentrations of Mg^2+^ and time of exposure. ^@,+,&^P<0.05, for differences over time at concentrations of 1, 2 and 3 mM, respectively. ^#^P<0.05, for differences among various concentrations of Mg^2+^ at each time point. *P<0.05, for differences from the control group at each time point (ANOVA).

The ALP activity and osteocalcin levels of osteoblasts exposed to the magnesium ions at the three concentrations were evaluated (Figures 2 and 3, respectively). All results showed that the stimulation action of 3 mM magnesium for 72 h was the highest. The ALP activity and osteocalcin levels of osteoblasts treated with magnesium ions increased with higher concentrations of magnesium ions and longer exposure time.

### GJIC in human osteoblasts

**Figure 4:**
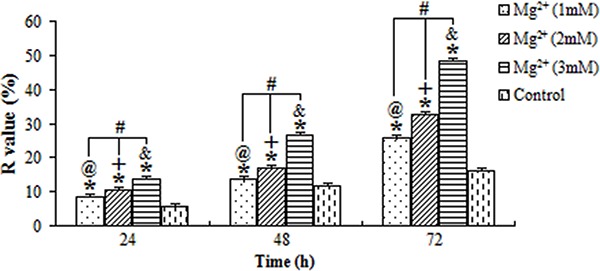
Average fluorescence recovery (R value) for osteoblasts treated with magnesium (Mg^2+^). The stimulation action was positively associated with the concentration of Mg^2+^ and the time of exposure. ^@,+,&^P<0.05, for differences over time at concentrations of 1, 2 and 3 mM, respectively. ^#^P<0.05, for differences among various concentrations of Mg^2+^ at each time point. *P<0.05, for differences from the control group at each time point (ANOVA).

The ratios of fluorescence recovery (R) of cells exposed to magnesium ions at the concentrations of 1, 2 and 3 mM for 24, 48 and 72 h are shown in Figure 4. Compared to the control group, the GJIC of osteoblasts was significantly promoted by the magnesium (P<0.05). Meanwhile, the highest R-value was found in cells exposed to 3 mM magnesium for 72 h, whereas the lowest was found in cells exposed to 1 mM magnesium for 24 h, suggesting that the R-value of cells was also positively associated with the concentration of magnesium ions and the duration of exposure.

## Discussion

Recently, magnesium (Mg) alloys or Mg-coated surfaces of metallic substrates were introduced in medical applications (12,13). A few studies have reported an increase in the attachment and functions of osteoblasts on the Mg surface (14,15). Moreover, Mg is the fourth most abundant mineral in the human body, the second most abundant in intracellular medium and the most abundant in cartilage and bone tissue during the initial stages of osteogenesis (16). Some studies have demonstrated that magnesium ions play a momentous role in osteogenesis and bone remodeling (17-20). However, because of the complexity of the mechanisms regulating bone remodeling, the experimental and mechanistic studies based on *in vivo* murine genetic and pharmacological models had their limitations to some extent. Furthermore, previous studies (4,6,9) based on osteoblastic cell lines mainly derived from animal or osteosarcoma have provided a better understanding of the action that was implicated in the control of osteoblasts activity and thus bone formation. Thus, we used the normal human osteoblast cell line in this study, which markedly improved the reliability of the research.

This study is unique in that it comprehensively investigated the dose-dependent effects of Mg ions on human osteoblasts, looking specifically at cellular proliferation and function. The effects of Mg ions at different concentrations on osteoblasts were also compared. The results showed that Mg ions increased not only cell viability but also cell differentiation. We used ALP activity and osteocalcin as markers for osteoblastic differentiation. All groups treated with Mg had dose-dependent up-regulation of ALP activities and osteocalcin levels compared with the control group. Several researchers have studied Mg actions on osteoblastic cell proliferation, differentiation, or function. Our results were in line with the study by Yang et al (21), who evaluated the effects of Mg alloys on the osteogenic differentiation of human bone marrow-derived stromal cells (hBMSCs) and found that Mg ion in the extracts, which was a result of the corrosion of Mg alloys, stimulated the viability and osteogenic differentiation of hBMSCs. Abed and Moreau (9) also demonstrated that the influx of Mg ions enhanced cell migration and stimulated the gene expression of *TRPM7* channels in human osteoblast MG-63 cells. The stimulatory action of Mg ion on human osteoblasts indicated that it was involved in modulating bone metabolism in some way. However, the mechanism of action of Mg needs to be further investigated.

Many studies have been conducted to evaluate the osteogenic action of Mg, but few have examined the effects of various concentrations of these ions. In the current study, osteoblasts viability was positively associated with the concentration of Mg ions and the duration of exposure. Higher concentrations had greater effects on osteoblasts viability. Our findings are in agreement with the experimental study performed by Yang et al. (21), which indicated that ≤10 mM concentration of Mg ion in the extracts enhanced the viability and osteogenic differentiation of hBMSCs. Another recent study found that the increase of intracellular Mg concentration could stimulate the proliferation and migration of human osteoblast MG-63 cells (9). In contrast to our findings, however, Leidi et al. (22) reported that high Mg levels impaired osteoblast activity, which might therefore contribute to bone disease. Similarly, Kircelli et al. (23) found that higher Mg levels prevented the process of differentiation of bovine vascular smooth muscle cells into osteoblast-like cells and inhibited expression of osteogenic proteins, apoptosis and further progression of already established calcification. The different results in our study could be due to different study protocols, such as the various cell lines used in studies, the duration of culturing, the diluents for Mg, and methods used to evaluate cell viability. Although we originally investigated three concentrations of Mg ions, the most effective dose of action is still not clear, and an extended dose range needs to be further tested.

Collectively, these results suggest that the Mg ion stimulated osteogenesis in human osteoblasts. Previously, it was observed that Mg depletion in rat and/or mouse increased osteoclastic bone resorption and inhibited bone formation. Also, Mg deficiency in humans has been associated with altered osteoblastic differentiation, reduced bone formation and the development of osteoporosis (8). These diverse data supported the hypothesis that Mg contributed to the maintenance of bone mass by regulating osteogenic activity in osteoblast. However, the mechanism for this action is still unclear. Signal factors, depending on their properties and their impact on messenger substance, can affect cell differentiation and bone formation. Furthermore, signal transmission has an essential role in cell activation. GJIC mediated by connexins, in particular connexin 43 (Cx43), plays important roles in regulating signal transmission among different bone cells, and thereby regulates development, differentiation, modeling and remodeling of the bone. Furthermore, osteocytes utilize GJIC to coordinate bone remodeling in response to anabolic factors and mechanical loading. These transmembrane channels allow continuity of cytoplasm and mediate the transfer of molecules between communicating cells (24-26). The biological importance of the communication mediated by connexin-forming channels in bone development is revealed by the low bone mass and osteoblast dysfunction in the Cx43-null mice and the skeletal malformations observed in occulodentodigital dysplasia caused by mutations in the Cx43 gene (27-30). However, there are no available data on the effects of the Mg ion on GJIC between osteoblasts so far.

The novelty of the current work is that we confirmed the stimulatory effect of Mg ion on GJIC in osteoblasts with the FRAP technique. The R-value of cells increased with increasing Mg ion concentrations and exposure time, suggesting that Mg ion regulated osteoblast activity via promotion of the GJIC between cells. This is further confirmed by the role of Mg in the mediation of osteoblast proliferation, ALP and osteocalcin production. Our opinion is that gap junctional communication may serve as a means by which osteoblasts could function in synchrony and propagate locally generated signals throughout the skeletal tissue. Thus, we argue that Mg may enhance the GJIC between osteoblasts, and then promote the transfer of molecules between communicating cells. In this way, it would improve the response of osteoblasts to various stimulating signals. These data provide further support for the hypothesis that stimulatory effects of Mg ion on osteoblasts and osteogenesis activity are not only due to the direct binding and activation of their receptors, but also due to a direct action on the GJIC that coordinates bone remodeling.

Furthering our understanding of the mechanisms underlying the involvement of the Mg ion in the control of bone remodeling could lead to the development of therapies to improve bone mass in patients with diseases such as osteoporosis. However, the results reported here are preliminary. Many factors, such as the specificity and effective concentration of the Mg ion that are closer to the physiological state, should be taken into account in future work.
